# Understanding central sensitization for advances in management of carpal tunnel syndrome

**DOI:** 10.12688/f1000research.22570.1

**Published:** 2020-06-15

**Authors:** César Fernández-de-las-peñas, José L Arias-Buría, Ricardo Ortega-Santiago, Ana I De-la-Llave-Rincón

**Affiliations:** 1 Department of Physical Therapy, Occupational Therapy, Rehabilitation and Physical Medicine, Universidad Rey Juan Carlos, Alcorcón, Spain; 2 Cátedra Institucional en Docencia, Clínica e Investigación en Fisioterapia: Terapia Manual, Punción Seca y Ejercicio Terapéutico, Universidad Rey Juan Carlos, Alcorcón, Madrid, Spain

**Keywords:** Carpal tunnel, pain, sensitization, nociceptive pain.

## Abstract

Carpal tunnel syndrome is the most common nerve compression disorder of the upper extremity, and it is traditionally considered a peripheral neuropathy associated with a localized compression of the median nerve just at the level of the carpal tunnel. Surgery and physiotherapy are treatment approaches commonly used for this condition; however, conflicting clinical outcomes suggest that carpal tunnel syndrome may be more complex. There is evidence supporting the role of peripheral nociception from the median nerve in carpal tunnel syndrome; however, emerging evidence also suggests a potential role of central sensitization. The presence of spreading pain symptoms (e.g. proximal pain), widespread sensory changes, or bilateral motor control impairments in people presenting with strictly unilateral sensory symptoms supports the presence of spinal cord changes. Interestingly, bilateral sensory and motor changes are not directly associated with electrodiagnostic findings. Other studies have also reported that patients presenting with carpal tunnel syndrome exhibit neuroplastic brainstem change supporting central sensitization. Current data would support the presence of a central sensitization process, mediated by the peripheral drive originating in the compression of the median nerve, in people with carpal tunnel syndrome. The presence of altered nociceptive gain processing should be considered in the treatment of carpal tunnel syndrome by integrating therapeutic approaches aiming to modulate long-lasting nociceptive barrage into the central nervous system (peripheral drive) and strategies aiming to activate endogenous pain networks (central drive).

## Introduction

Carpal tunnel syndrome (CTS) is the most prevalent nerve compression disorder of the upper extremity, and it is generally associated with a localized compression of the median nerve in the carpal tunnel area. Although epidemiological data vary depending on the diagnostic criteria used, CTS has an incidence rate of 1.8/1,000 ^1^ and a prevalence rate ranging from 6.3 to 11.7% in the general population ^2^. Since people affected by CTS are usually active workers, this condition is associated with substantial healthcare costs and economic burden ^3^. Therefore, a better understanding of the potential underlying mechanisms of CTS could improve therapeutic strategies. This paper discusses current theories combining the presence of peripheral nociceptive barrage with the presence of altered nociceptive central processing and its repercussions for the potential management of CTS. The current paper is an updated version of a previous review on this topic ^4^.

This review will try to answer the following questions: 1. is CTS just a localized peripheral entrapment of the median nerve? 2. What are the underlying mechanisms behind the clinical manifestations of CTS? 3. Does the presence of altered nociceptive gain potential have repercussions on treatment approaches in CTS?

## Understanding carpal tunnel syndrome

### Spreading pain symptoms in carpal tunnel syndrome

The most common symptoms usually experienced by patients with CTS include pain and/or paresthesia in areas innervated by the median nerve, i.e. the thumb, index, and/or middle fingers. Symptoms can be worse during activities involving the hand/wrist but also at night. It seems that paresthesia, but not pain, is the symptom most commonly associated with neurophysiological damage of the median nerve ^5^.

It is commonly seen in clinical practice that patients with CTS exhibit symptoms not only in those areas innervated by the median nerve but also in extra-median nerve areas. This was confirmed by Zanette *et al.*, who observed that 35% of patients with CTS exhibit a glove distribution of their symptoms, whereas 5% exhibit an ulnar distribution ^6^. The same authors also reported that 45% of CTS sufferers also report pain in proximal areas of the upper extremity including the elbow or the shoulder ^7^. A more recent study, using new electronic software for analyzing pain extent, found that 88% of women with CTS showed extra-median pain symptoms ^8^. The presence of spreading symptoms is a clinical manifestation of neuropathic but also nociceptive pain involving sensitization mechanisms and plasticity ^9^. This hypothesis is confirmed by the fact that extra-median symptoms are not associated with electrical nerve conduction data, suggesting an association between neuropathic and nociceptive pain with expansion of receptive fields of central neurons in CTS ^10^.

### Peripheral and central sensory changes in carpal tunnel syndrome

‘Sensitization’ is a term describing the changes in nociceptive neurons. It is mainly grouped into peripheral/central sensitization, and it is present in several musculoskeletal and neuropathic chronic pain conditions ^11, 12^. ‘Peripheral sensitization’ refers to increased responsiveness and reduced threshold of peripheral nociceptors; it is usually associated with the release of endogenous algogenic substances and neurogenic inflammation. ‘Central sensitization’ is defined by the International Association for the Study of Pain (IASP) as an increased responsiveness of the nociceptive neurons in the central nervous system to their normal or subthreshold afferent input, which may include increased responsiveness due to dysfunction of endogenous pain control systems ^13^. The relationship between peripheral and central sensitization is well established. It is accepted that the presence of long-lasting and prolonged nociceptive peripheral inputs induces plastic changes in the central nervous system; therefore, central sensitization seems to be a dynamic process that is dependent on peripheral nociception ^11, 12^. In some patients, once central sensitization has been established, minimal peripheral nociception can be required to maintain this process and non-nociceptive inputs might also contribute to the subsequent pain and mechanical allodynia observed in this situation. Nevertheless, central sensitization is mainly driven by peripheral impulses. In such a scenario, in people with CTS, the entrapment of the median nerve at the carpal tunnel represents the peripheral input towards the central nervous system. Gracely *et al.* proposed this model for neuropathic pain in 1992 where peripheral nociception from an entrapped nerve could lead to neuroplastic changes in the central nervous system ^14^. In CTS, the neurogenic inflammation of the median nerve induced by its entrapment can act as a peripheral driver for the gradual sensitization of nociceptive pathways. In fact, Truini *et al.* found that affectation of both nociceptive and non-nociceptive fibers contributed to the different symptoms experienced in CTS ^15^.

The peripheral component of entrapment neuropathies such as CTS is supported by current literature ^16^ and will not be further discussed in this text. In the last decade, different studies have investigated the presence of central sensitization by assessing widespread sensory disorders throughout quantitative sensory testing in CTS. Fernández-de-las-Peñas *et al.* found that women with strictly unilateral CTS symptoms exhibit widespread pressure pain sensitivity over the radial, ulnar, and median nerves, the cervical spine, and the tibialis anterior muscle, suggesting widespread hypersensitivity to pressure pain ^17^. Widespread pressure hypersensitivity is a manifestation of the sensitization of central pathways; however, pressure pain hypersensitivity has also been found to be heterogeneously distributed in the symptomatic hand, supporting the relevant role of the peripheral input ^18^. Similar results were also found by Zanette *et al.*, who observed that CTS patients with extra-median symptoms exhibited generalized pressure hyperalgesia and enhanced wind-up ^19^. Interestingly, widespread pressure pain hyperalgesia was not associated with the electrodiagnostic findings, since women with minimal, moderate, or severe CTS exhibit similar widespread sensory changes ^20^. These results support the notion that widespread hyperalgesia to pressure pain can be a common manifestation of CTS from the beginning of symptom onset. In line with this hypothesis, Tampin, Vollert, and Schmid found that individuals with a peripheral neuropathy such as CTS exhibit similar sensory changes to those with a proximal (cervical) radiculopathy, although pain profiles were different ^21^. Nevertheless, Schmid *et al.* did not observe widespread pressure hyperalgesia in patients with CTS without concomitant neck pain symptoms, suggesting that groups of patients with CTS could exist and that the observed widespread sensory changes can be more related to the presence of extra-median/proximal symptoms ^22^. The hypothesis of different subgroups of patients with CTS has been supported by a study identifying a subgroup of women with CTS with higher bilateral widespread pressure hyperalgesia based on the presence of a cluster of symptoms ^23^. Therefore, it is possible that CTS can be peripherally mediated in some patients but more centrally mediated in others.

The presence of central sensitization in CTS is also supported by imaging studies. 

Tecchio *et al.* found that patients with CTS with symptoms of glove distribution exhibited an enlargement in cortical representation and neuroplastic changes in the somatosensory cortex compared to those individuals with just median nerve distribution symptoms ^24^. Different studies have also shown larger contralateral sensorimotor cortical representation ^25^ and smaller cortical source separation ^26^ of the second/third digits in patients with CTS as compared to controls. All of these studies hypothesized that long-lasting paresthesia/pain symptoms (peripheral drive) can promote blurring of median nerve-innervated digit representations through neural mechanisms (central drive) ^25, 26^. There is also current evidence showing the presence of reduced endogenous inhibition and increased pain facilitation in patients with CTS ^27^. In conclusion, long-lasting peripheral nociceptive stimuli originating in the median nerve could lead to neuroplastic changes in the central nervous system at both spinal cord and brainstem levels.

### Motor output changes in carpal tunnel syndrome

It is interesting to consider that sensitization mechanisms and sensory loss also have output manifestations. Motor control disturbances may be a perpetuating factor for pain since pain-related fear or avoidance behaviors can also promote negative attitudes. In addition, altered motor control patterns could reflect the reorganization of motor control strategies in the central nervous system, as sensory neuroplastic changes in the somatosensory cortex result in decreased fine motor control ^28^. The presence of bilateral deficits in fine motor control and pinch grip force in women with strictly unilateral CTS supports this hypothesis ^29^. Additionally, these bilateral deficits in fine motor control and pinch grip force were not associated with either median nerve damage or the presence of unilateral or bilateral symptoms ^30^. Data suggest that motor output disorders are present from the onset of symptoms and could be related to neuroplastic changes in the cortex. Nevertheless, it is probable that peripheral and central mechanisms can be involved at the same time in the motor control disturbances observed in CTS.

## Implications for clinical practice

Although CTS can be treated with conservative and surgical procedures, surgery continues to be the most common treatment approach proposed for these patients ^31^. In fact, both open and endoscopic surgical interventions provide similar clinical results ^32^. However, although surgery provides positive long-term results, it may not always be entirely successful ^33^, and the recurrence rate is estimated to be around 30% (from 5 to 57%) ^34^. There is great debate related to which patients with CTS should be treated with conservative or surgical treatment in the first instance ^35^. In fact, no consensus exists on which therapeutic option is the best for CTS. A recent meta-analysis has reported that the differences between surgery and conservative treatment are small ^36^. Interestingly, Jarvik *et al.* observed that 61% of patients with CTS attempted to avoid surgery ^37^. Conservative treatment for CTS could be preferred as the first-line option for patients with minimal or moderate CTS, whereas surgery could be the first-line approach for patients with more severe symptoms or when conservative treatment has failed.

It is important to consider that there is no established algorithm for determining which patients with CTS will benefit from conservative treatment ^38^, i.e. physiotherapy.

In fact, different meta-analyses reported inconclusive evidence for the use of different conservative interventions, such as low-level laser ^39^, ultrasound ^40^, or splinting ^41^. Other conservative techniques, e.g. nerve/tendon (neuro-mobilization) gliding exercises, have shown promising, but also limited, outcomes ^42^. Discrepancies in the effects of multiple and different management therapeutic strategies can be attributed to the fact that CTS is a heterogeneous syndrome. In such a scenario, the presence of sensitization mechanisms discussed in this text could provide a physiological explanation for those individuals with CTS who experience persistent symptoms despite apparently successful surgery.

A mechanism-based classification can help clinicians to understand that different signs and symptoms may reflect different underlying nociceptive gain mechanisms. The presence of sensitization mechanisms in people with CTS has potential implications for clinical practice, since it implies that, in some patients, more global, not just localized, interventions should be applied. According to current data, clinicians should identify if symptoms in a patient with CTS are mainly peripherally or centrally mediated. In patients with peripherally mediated symptoms, localized interventions targeting local tissue (i.e. median nerve entrapment at the carpal tunnel) should be provided. There is evidence supporting a peripheral effect of nerve/tendon gliding exercises in patients with CTS, since these exercises affect the biological integrity of the median nerve by increasing fluid dispersion and reducing intraneural edema ^43, 44^. In patients with centrally mediated symptoms, clinicians should incorporate therapeutic approaches directed at normalizing peripheral and central nervous system interactions (i.e. soft tissue-based techniques, nerve mobilizations, or pain neuroscience education) and not just localized interventions over the carpal tunnel. It is in this group of patients where medications targeting the central nervous system could be effective ^45^.

This clinical reasoning has been applied in a randomized clinical trial comparing the effects of manual therapy including desensitization maneuvers of the central nervous system with surgery in women with CTS ^46^. This promising clinical trial showed that the application of manual therapies following altered nociceptive gain reasoning exhibited better short-term (1- and 3-month) and similar long-term (6- and 12-month) effects on pain and function than surgery in women with CTS ^46^. In fact, the cost-effectiveness analysis of this trial also revealed better economic aspects in favor of manual physical therapy ^47^.

Nevertheless, the challenge facing clinicians is how to select the most appropriate intervention for each patient, who is likely to be slightly different in their presentation. To determine the most appropriate therapeutic strategy, considerations must be given to interpreting the clinical manifestations of these sensitization processes (peripheral and/or central) and to potential prognostic factors related to clinical outcomes. Nevertheless, although central sensitization has been related to poorer outcome in response to surgical or conservative treatment in musculoskeletal pain, this hypothesis is supported by small and exploratory studies, and no conclusion can be made for this assumption ^48^. A recent study has reported that localized, but not widespread, pressure sensitivity was predictive of long-term clinical outcomes in a sample of women with CTS after the application of manual therapies including desensitization maneuvers of the central nervous system ^49^. These preliminary results support the clinical reasoning that not all patients with CTS respond to the same therapeutic approach and subgroups of patients could exist.

## Conclusion

This updated review of underlying mechanisms of CTS found that there is evidence supporting the presence of central sensitization mechanisms, potentially mediated by the peripheral nociceptive inputs from the median nerve, in patients presenting with CTS. The presence of central sensitization influences treatment prognosis and the response of patients with CTS to a particular therapeutic strategy. The current paper has proposed the existence of two main subgroups of patients: those with peripherally mediated CTS and those with centrally mediated CTS. Clinicians should apply therapeutic strategies based on current knowledge on pain neuroscience and according to the clinical presentation and predominant identified mechanisms in these patients.
